# The Spiritual Dimensions of Friendship

**DOI:** 10.1093/geroni/igaa057.2266

**Published:** 2020-12-16

**Authors:** Andy Achenbaum

**Affiliations:** Texas Medical Center, Houston, Texas, United States

## Abstract

It was the spiritual dimensions of my friendship with Vern Bengtson that I treasure most. Vern was always willing to discuss the dark sides of himself and to listen to my spiritual pain. His empowering way of advancing the meanings of aging were a spiritual gift. This presentation will address the value of spiritual friendship in human aging. Part of a symposium sponsored by the Religion, Spirituality and Aging Interest Group.

